# Some War Problems in Food

**Published:** 1917-02-24

**Authors:** 


### SOME WAR PROBLEMS IN FOOD.

### The Bread Ration.

It will come as a surprise to many hospital managers that half a pound of bread a day, with an extra half-pound weekly, say, of cake, is by universal consent considered a sufficient allowance for each person in a mixed diet. In days of ignorance and extravagance the daily allowance of bread for each member of the nursing and household staff was commonly a pound. Then it began to be discovered that it was more economical not to allowance the household, but to let all eat as much bread as they liked. And the consumption of bread went down by leaps and bounds. Referring to "Hospital Expenditure—The Commissariat," it will be found that under this improved system the actual quantities consumed in certan well-managed hospitals were pretty exactly what the Government has now imposed as a war ration. With bread at a penny a pound, as in the old days, the war ration of 4 lbs. a week works out at an average cost of 17s. 4d. per head per annum. Now, the average cost of bread per head per annum in 1893 was 17s. at Hull Royal Infirmary, and 18s. at Birmingham Queen's Hospital. At the Leeds General Infirmary it was 16s., at the Bradford Infirmary 17s., and at the York County Hospital 18s., but the three latter institutions enjoyed a private bakehouse, which should considerably reduce the cost. Flour, oatmeal, barley, cake, etc., are included in these totals, as in the present-day ration.

The actual quantity of bread consumed on an average yearly by each member of the household, including nursing staff, in certain institutions, was found to be as follows:—Guy's Hospital, average per patient, 239 lbs.; per member of household, 245 lbs.; Birmingham General Hospital, average per patient, 224 lbs., per member of household, 162 lbs.

| | lbs. | Cost. |
|---|---|---|
| Bristol General Infirmary ... ... | 219 | £0 19s. |
| Royal Sussex County Hospital ... | 243 | £1 4s. |
| Radcliffe Infirmary, Oxford, ... | 263 | £1 0s. |
| Belfast Royal Victoria Hospital... | 294 | £1 6s. |
| Norfolk and Norwich Hospital ... | 295 | — |

The ration of 4 lbs. a week totals but 208 lbs. in a year, even without reckoning anything off for cake or puddings. So, taking half a pound to be a very fair quantity (and there are few who do not find it sufficient), it is evident that in some of the above institutions the unseen mouths which consume so much in houses large and small must have been busy. These unseen mouths are to be discovered, for ever insatiable, in the pig-tub and waste-pail. Even in establishments where much stress is laid upon economy they may easily and without being noticed nibble away an average amounting to a fourth of the Government ration, or 1 lb. of bread per head per week.

The task for managers to tackle in the present emergency is to capture that extra pound of bread per week on its way to the waste-pail, and divert it to its proper use.

The average daily allowance of bread suggested by the author of the "Commissariat" as fair is 12 oz. per head, or 273 lbs. a year. In the light of further knowledge it now seems as if this would leave just that 1 lb. a week for the "invisible mouth" which the nation is now determined to close.

